# Enhanced Fluorescence of N-Acetyl-β-D-Glucosaminidase Activity by ZnO Quantum Dots for Early Stage Mastitis Evaluation

**DOI:** 10.3389/fchem.2019.00754

**Published:** 2019-11-08

**Authors:** Narsingh R. Nirala, Giorgi Shtenberg

**Affiliations:** Institute of Agricultural Engineering, ARO, The Volcani Center, Bet Dagan, Israel

**Keywords:** biomarker, enhanced fluorescence, mastitis, N-acetyl-β-D-glucosaminidase, quantum dots

## Abstract

Recurrent mastitis events are the major cause of annual revenue losses in the dairy sector resulting in decreased milk yield, escalading treatment costs and increased health risk of the entire herd. Upon udder inflammation, several biomarkers are proportionally secreted to its severity onto the blood circulation and consequently into milk (upon breached blood-milk barrier). N-acetyl-β-D-glucosaminidase activity is widely used mastitis indicator in milk, offering simple means of differentiation between healthy quarters from those with subclinical or clinical severity. Herein, we demonstrate fluorescence signal amplification concept for sensitive clinical status discrimination. Tetraethyl orthosilicate coated zinc oxide quantum dots were employed within the conventional N-acetyl-β-D-glucosaminidase activity assay. Under the experimental conditions, a profound non-radiative energy transfer occurred between quantum nanomaterials onto enzymatic fluorescent products resulting in intensified emission of the latter, over 11-folds, in comparison to nanoparticle-free assay. Overall, the fluorescence intensities were proportionally related to zinc oxide quantum dots surface coverage and concentration, SCC values and influenced by the causing bacteria (i.e., *Streptococcus dysgalactiae* and *Coagulase-negative Staphylococci*). Finally, the presented proof-of-concept offers an efficient, simple, cost-effective fluorescence signal amplification for early stage mastitis identification, offering means to diagnose the severity of the associated diseases and hence deducing on animals' clinical status.

## Introduction

Recurrent mastitis events are the major cause of annual revenue losses in the dairy sector resulting in decreased milk yield, escalading treatment costs and increased risk of culling or even death (Ferrero et al., 2014; Hovinen et al., 2016). Traditionally, mastitis detection depends on the efficacy and reliability of conventional techniques, which are designed to measure somatic cell counts (SCC), detect causative pathogenic bacteria and reveal inflamed status associated with secreted biomarkers (Riffon et al., 2001; Sears and Mccarthy, 2003; Viguier et al., 2009; Mujawar et al., 2013). These biomarkers include: haptoglobin, serum/milk amyloid A and N-acetyl-β-D-glucosaminidase (NAGase) and others, which are secreted onto plasma or milk upon inflammation, infection or trauma (Viguier et al., 2009; Kalmus et al., 2013). NAGase is a prominent inflammatory indicator widely used in correlation to SCC values in milk (Pyörälä et al., 2011). This intracellular lysosomal glycosidase is released from damaged epithelial cells of the mammary tissue and is associated with cell lysis, hence indicating tissue destruction (Hovinen et al., 2016). NAGase activity levels reflect the degree of induced mastitis, thus can separate healthy quarters from those with subclinical or clinical severity (Pyörälä et al., 2011). Numerous case studies have utilized this biomarker for discriminating the degree of inflammation in cows, ewes, goats and camels (Guliye et al., 2002; Kalmus et al., 2013). The biochemical activity is affected by the causing bacteria type, days in milk, parity and animal's milking phase (Hovinen et al., 2016). NAGase activity determination in whole milk samples was first described by Kitchen et al. that has inspired an extensive research within this field (Kitchen et al., 1984). The methodology includes the digestion of non-fluorogenic substrate 4-methylumbelliferyl N-acetyl-β-D-glucosaminide (4-MUAG) in acidic conditions to the release of 4-methylumbelliferone (4-MU) fluorophore. Despite the prominent advantages of the assay, i.e., efficient, cost-effective and simple, fluorescence (FL) based detection mechanism suffers from fundamental drawback associated with low quantum efficiency and photobleaching, which may affect the sensitivity threshold while differentiating various milk classes (Jeong et al., 2018). Additionally, the technique is mostly based on off-line monitoring (relying on expensive laboratory equipment) with low frequency data sampling and delay upon results availability.

Various quantum nanomaterials (quantum dots, QDs) have been applied for biosensing application utilizing their unique optoelectronic properties, e.g., high quantum yield, quantum size effects, broad absorption, and tunable emission spectrum, providing superior sensitivity for real world sample analysis (Bauermann et al., 2006; Wu et al., 2007; Patra et al., 2009; Gu et al., 2011; Sharma et al., 2013). Zinc-oxide (ZnO) is versatile semiconducting material presenting a wide band-gap (3.37 eV) and rather large exciton binding energy (60 meV), resulting in profound room temperature exciton state stability (Wang et al., 2018). ZnO is a biocompatible, eco-friendly, non-toxic and low-cost material produced by diverse wet chemical processes, specifically sol-gel reaction (Patra et al., 2009). The stability of colloidal ZnO nanomaterials in aqueous media limits their long-term practical utilization as they tend to physically expand and agglomerate during storage, revealing excessive FL quenching (Wu et al., 2007). Thus, surface modification is applied to control nanoparticle functionality, size, morphology, thermodynamic stability, unwavering luminescence, or emission levels by employing different capping agents (such as: polyvinylpyrrolidone, amines, aliphatic thiols, and silanes) (Patra et al., 2009). Several research groups have been engaged in linking ZnO nanostructures morphologies to the FL enhancement across the visible-light spectrum for practical usage. Wang et al. presented a detailed insight of ZnO nanorods and nanoflowers structure property dependence to influence FL enhancement factor by varying size and shape (Wang et al., 2018). Zhao et al. developed ZnO-QDs (ca. ~ 5 nm) modified with (3-aminopropyl)triethoxysilane using a rapid two-step procedure, for direct detection of dopamine in human serum for clinical analysis (Zhao et al., 2013). The dopamine molecules adsorbed on ZnO-QDs outer surface hindered the electron transfer reaction resulting in significant FL quenching. Under optimized conditions the bioassay presented a wide linear range from 0.05 to 10 μM, with limit of detection (LOD) of 12 nM and high quantitative recovery values of 99-101%. Another assay have utilized an imine linked receptor for sensitive detection of cobalt ions by quenching the FL intensity of 13.8 nm hexagonal wurtzite nanostructures (Sharma et al., 2013). The ratiometric FL sensor recognizes the target molecules in a semi-aqueous solvent system (dimethyl sulfoxide/H_2_O), while presenting LOD of 0.4 nM. Gu et al. developed ZnO-QDs dual detection (electrochemical and FL) of carbohydrate antigen 19-9 as a label for pancreatic cancer by standard sandwich-immunoassay (Gu et al., 2011). The biorecognition event of the target antigen by labeled QDs was transformed into amplified signal measured by square wave stripping voltammetry and intrinsic photoluminescence. Both assays demonstrated a wide dynamic range of 0.1–180 U mL^−1^ and 1–180 U mL^−1^, LOD of 0.04 U mL^−1^ and 0.25 U mL^−1^ for electrical and optical detection, respectively. The high ZnO-QDs performance level offered sufficient stability, reliability and reproducibility that can be easily adapted for other cancer biomarkers circulated in serum/plasma.

In the current study, we applied a similar optical amplification sensing concept for sensitive discrimination of subclinical and clinical mastitis using silica coated ZnO-QDs (ZnO-QDs-SiO_2_) within the conventional NAGase activity assay. The optical properties of the synthesized nanomaterials were fully characterized, considering the optimal catalyst concentration added onto the assay. The enzymatic reaction products were studied in the presence of the quantum nanomaterial with respect to a conventional NAGase assay (omitting ZnO-QDs). The SCC levels and the mastitis inducing pathogenic bacteria influence on NAGase activity were also evaluated with respect to the sensing performance of a conventional assay. Overall, the efficiency of the presented proof-of-concept was evaluated to offer means of severity diagnosis of the associated diseases and implying on udder health status (mastitis recovery prognosis).

## Materials and Methods

### Materials

Zinc nitrate hexahydrate (98%), potassium hydroxide, sodium citrate dihydrate, hydrochloric acid (HCl), methanol and tetraethyl orthosilicate (TEOS, 98%) were obtained from Merck. 4-MUAG, 4-MU and analytical grade buffers were supplied by Sigma-Aldrich. All reagents unless otherwise mentioned were analytical reagent grade. Milli-Q water (18 MΩ cm) was used for all the denoted experiments.

### Synthesis of ZnO-QDs-SiO_2_

ZnO-QDs were synthesized according to a previous study (Patra et al., 2009). Briefly, 0.1 M potassium hydroxide was dropwise added onto 0.1 M of zinc nitrate hexahydrate solution (both dissolved in methanol) under continuous stirring. The solution was left stirring for 1 h to homogenize its content, while maintaining the pH at ~10, resulting in ZnO-QDs. Next, 250 μL of TEOS was added onto the product solution (optimized volumetric condition within the range of 0–250 μL) followed by 500 μL distilled water addition to control the particle surface coverage by the silica molecules, thus producing ZnO-QDs-SiO_2_. The colloidal solution was thoroughly cleaned by centrifuging (10,000 rpm for 10 min), washed with methanol three times. Finally, the precipitated product were redisposed in milli-Q water (producing concentration of 12.8 mg mL^−1^) for further study.

### Apparatus and Measurements

The quantum nanomaterials were characterized by UV-VIS absorption and FL emission spectra recorded with multimode microplate reader (Varioskan™ LUX by Thermo Scientific). Elemental mapping was performed by energy-dispersive X-ray spectroscopy (EDX) using MIRA3 field-emission SEM microscope (TESCAN) coupled with EDX detector at an acceleration voltage of 10 kV. Transmission electron microscopy (TEM), JEOL JEM-1400, was applied for studying structural morphology and size, operated at 120 kV. Attenuated total reflectance Fourier transform infrared (ATR-FTIR) spectroscopy was used to further confirm surface modification using Thermo Scientific Nicolet iS50, as previously noted (Massad-Ivanir et al., 2011).

### Milk Samples

Samples were collected by trained personnel from specific quarters of Holstein cows (Volcani Center) representing normal (healthy), spontaneous subclinical or clinical mastitis, as previously described (Nirala et al., 2019). Animal experiments were approved by the Institutional Animal Care Committee of ARO, The Volcani Center, Bet Dagan, Israel and followed its ethical guidelines (approval number 838/119IL).

### NAGase Activity Determination

NAGase activity was performed according to a modified protocol (Hovinen et al., 2016). Briefly, each microplate well plate was treated with 30 μL of milk, 40 μL of 2.25 mM 4-MUAG substrate or a range of 4-MU product concentrations (0–20 μM) in citrate buffer (0.25 M, pH ~ 4.6) and 100 μL of optimized quantum material concentration. Various ZnO-QDs-SiO_2_ concentrations (0, 1.28, 2.56, 3.88, 5.12, and 12.80 mg mL^−1^) were used to examine the optimal nanomaterial content. All reactions were placed on an automatic shaker for 60 s followed by end-point kinetics FL examination (E_x_/E_m_ of 365/450 nm) fixed for 8 min by the dedicated multimode microplate reader.

### Statistical Analysis

Statistical analysis was performed using a Student's *t*-test with a minimum confidence level of 0.05 for statistical significance and assuming unequal sample sizes and unequal variance. All values are reported as the mean ± standard deviation (*n* ≥ 3), unless otherwise stated.

## Results and Discussion

### Optical and Surface Characterization of Quantum Nanomaterials

**Figure 1:**
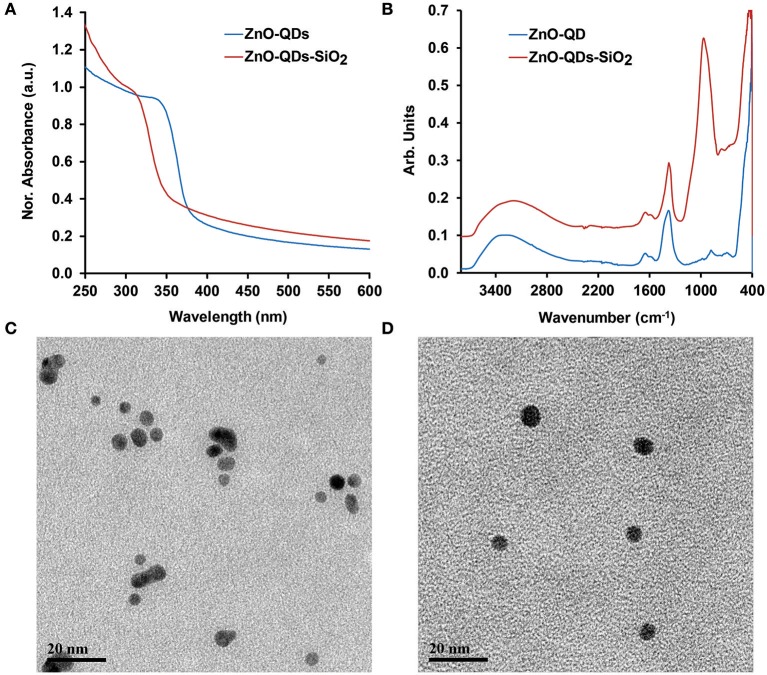
**(A)** UV-VIS and **(B)** ATR-FTIR spectra obtained before and after TEOS modification of ZnO-QDs; TEM images of **(C)** ZnO-QDs; and **(D)** ZnO-QDs-SiO_2_.

TEOS capped ZnO-QDs were synthesized by a modified soft chemical reaction using zinc nitrate hexahydrate as a precursor (Patra et al., 2009; Znaidi, 2010). TEOS is a widely used alkoxyl silane applied on various nanomaterials and films (Hagura et al., 2011; El-Naggar et al., 2017; Fang et al., 2017; Watermann and Brieger, 2017). The terminal end groups were hydrolyzed in aqueous surrounding and reacted with hydroxyl groups on the exterior of ZnO-QDs surface (Wu et al., 2007). The modification was performed at a controlled manner, as the residual three alkoxyl groups may polymerize with neighboring alkoxyl molecules, resulting in a thick non-uniform layer. Thus, the optical properties of bare and silica coated ZnO-QDs were characterized by UV-VIS spectroscopy, Figure 1A. The absorbance spectra show a characteristic shoulder band edge at 338 nm which is blue shifted toward 308 nm upon TEOS addition for ZnO-QDs and ZnO-QDs-SiO_2_, respectively. The blue shift is mainly ascribed to the change in the produced nanomaterial particle diameter and an increase in the bandgap energy values upon silane modification (Wu et al., 2007; Patra et al., 2009). The presence of silica coverage was further examined by ATR-FTIR studies shown in Figure 1B. The unmodified ZnO-QDs IR spectrum shows characteristic Zn–O stretching vibration peak at 431 cm^−1^, absorption peaks at 881 and 1,378 cm^−1^ due to N-O vibration of the initial nitrate precursor, 1,649 cm^−1^ peak of H_2_O bending vibration (structural water of the precursor) and a broad peak around 3,274 cm^−1^ accredited to the stretching vibration of O-H residues (Newman and Jones, 1999; Bauermann et al., 2006; Biswick et al., 2007). The TEOS ZnO-QDs modification is characterized by the addition of a new peak to the acquired spectrum, presenting Si-O vibration peak at 978 cm^−1^ (Patra et al., 2009). The additional absorption peak of ZnO-QDs-SiO_2_ spectrum suggest outer silica shell formation. EDX detector coupled to SEM was also employed to further characterize the resulting silica coverage. Supplementary Figures S1A,B depict elemental analysis data (spectra and relative atomic percentage) of ZnO-QDs and ZnO-QDs-SiO_2_, respectively. Indeed, significant increase in oxygen and silicon elements are shown for the TEOS modified nanomaterials, while the initial zinc values are decreased. These results are in agreement with UV-VIS and ATR-FTIR experiments results, indicating silica coverage. Finally, representative TEM images of ZnO-QDs and ZnO-QDs-SiO_2_ are shown in Figures 1C,D, respectively. Both images depict spherical shape particles, which are mildly increased in size following TEOS modification from 5.0 ± 0.6 nm to 7.5 ± 1.0 nm. It should be noted that upon TEOS treatment, the time depended nucleation and precipitation of neighboring ZnO-QDs are inhibited (restricting their growth and aggregation), and thus stabilizes the colloidal aqueous solution formation by the capping mode (Patra et al., 2009; Zhao et al., 2013).

### FL Amplification Studies

**Figure 2:**
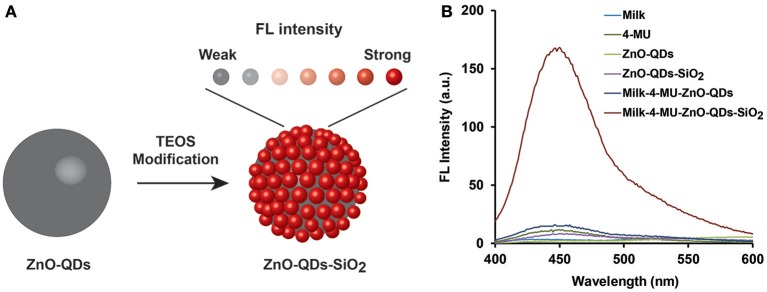
**(A)** A schematic illustration of FL signal amplification by modifying ZnO-QDs with TEOS molecules; **(B)** emission spectra of NAGase catalytic reaction products (20 μM 4-MU) in buffered and milk media with and without stock ZnO-QDs and ZnO-QDs-SiO_2_.

The FL enhancement of 4-MU product was characterized in diverse conditions to elucidate the emitted assay radiation amplification in real-life sensing conditions. A profound non-radiative energy transfer reaction is expected under the experimental conditions from the quantum nanomaterial onto the fluorophore molecules, thus leading to enhanced emission of the latter upon short-range intrinsic molecular interaction, as schematically illustrated in Figure 2A (Aslan et al., 2008; Wang et al., 2018). However, it should be noted that FL quenching effect may also occur, related to the reduction of the evanescent electric field around the surface of ZnO structure at non-optimal molecular distance, resulting in insignificant quantum yield (Gontero et al., 2017). Therefore, optimized TEOS coverage of bare ZnO-QDs was examined for maximal fluorescence enhancement effect (Ren et al., 2015). The latter could be achieved through sufficient overlap between the excitation spectra of the quantum material and the NAGase assay fluorophore (E_x_ of 365 nm). Supplementary Figure S2A shows the excitation spectra of the different TEOS volumes (0, 50, 100, 150, and 250 μL) used for ZnO-QDs modification. The excitation peak is red shifted upon higher silica coverage from 323 nm of bare ZnO-QDs up to maximal TEOS volumetric content (250 μL), which overlaps with the NAGase assay conditions (E_x_ of 365 nm). Supplementary Figure S2B depicts the corresponding emission spectra of the different TEOS modification, while presenting maximal FL response at 540 nm for 250 μL of TEOS. It should be highlighted that insignificant FL values are shown for all TEOS experimental conditions at 450 nm (characteristic E_m_ of the NAGase assay). Thus, considering the optimal molecular distance for maximal FL signal, the synthesis conditions were set to 250 μL of TEOS. Figure 2B depicts the corresponding emission spectra of NAGase catalytic reaction product (i.e., 4-MU) in buffered and milk media with and without quantum nanomaterials (bare and silica coated, respectively). Insignificant emission values are obtained for solutions omitting the fluorogenic product (i.e., milk, stock solutions of ZnO-QDs and ZnO-QDs-SiO_2_), in comparison to the intensity of 20 μM 4-MU product. Moreover, in both cases, bare and silica coated ZnO-QDs analyzed in the presence of 4-MU within milk showed enhanced FL signals. Silica layered nanomaterials present a significant enhancement (>11-folds) in comparison to bare ZnO-QDs, in which the enhancement surpasses the quenching effect (Figure 2B). These results are in agreement to previous studies showing that silica coating of diverse nanomaterials enhance the emitted optical signal (Aslan et al., 2008; Abadeer et al., 2014; Planas et al., 2016). Therefore, based on these results, silica coated ZnO-QDs were used for the consecutive analysis (catalytic reaction of NAGase) within milk samples.

**Figure 3:**
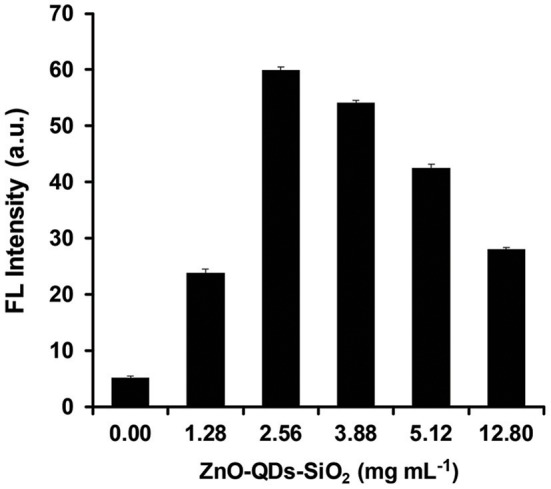
NAGase activity output in mastitic milk with respect to different concentrations of ZnO-QDs-SiO_2_. Data are reported as mean ± standard deviation (*n* ≥ 3).

Next, the optimized quantum material content within a given mass of reaction was studied trough NAGase catalytic activity by assessing the FL output within mastitic milk samples. It is expected that higher ZnO-QDs-SiO_2_ content will enhance the emitted signal due to higher accumulative electric field effect as increased surface area of nanostructured catalyst is used for each reaction (Aslan et al., 2008). Thus, different aqueous concentrations of ZnO-QDs-SiO_2_ were prepared (0, 1.28, 2.56, 3.88, 5.12, and 12.80 mg mL^−1^) from the resuspend stock nanomaterial. The FL intensities were evaluated by mixing 100 μL of each content set with 30 μL of mastitic milk sample and 40 μL of 2.25 mM 4-MUAG (NAGase substrate). The reaction was allowed to react for 8 min, while the emission values were recorded at 450 nm as an end-point kinetics. Figure 3 depicts the obtained FL values for the NAGase assay reaction products with respect to the catalyst content augmentation. Indeed, higher fluorogenic product (4-MU) is received with increasing quantum nanomaterial concentration from 10-fold and up to 5-fold stock solution dilution (1.28 and 2.56 mg mL^−1^, respectively). Above the latter concentration, a gradual decrease in the obtained FL signal is shown for 3.88, 5.12, and 12.8 mg mL^−1^ of ZnO-QDs-SiO_2_ (FL enhancement factors of 10.3, 8.1, and 5.4, respectively, in comparison to an assay omitting the addition of quantum nanomaterial). As previously noted, the resulting is accredited to the minute physical distance interactions of the resulting fluorophore product with the escalation in quantum nanomaterial concentration above a certain level, in which the quenching effect surpasses the FL enhancement (Ren et al., 2015). Taking into account the maximal FL enhancement (>11-fold) and the overall reagents depletion, the optimal ZnO-QDs-SiO_2_ concentration was set to 2.56 mg mL^−1^ for the residual milk analysis experiments.

### NAGase Evaluation Within Different Milk Samples

**Figure 4:**
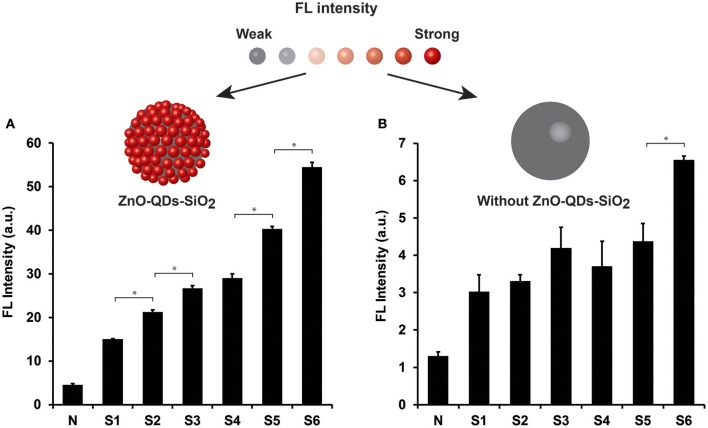
NAGase enzymatic activity output in different milk samples (normal, subclinical, and clinical mastitis) **(A)** with and **(B)** without ZnO-QDs-SiO_2_ addition onto the reaction assay solution. Top: the corresponding schematic illustration of FL signal amplification by TEOS modification of the ZnO-QDs. Data are reported as mean ± standard deviation (*n* ≥ 3). ^*^Statistically different (*t*-test, *p* < 0.05).

The analytical resolution of the enzymatic assay signal amplification was further investigated for a comprehensive milk quality spectrum evaluation. The analyzed milk samples were acquired from different animal udders and are summarized in Table 1. *Coagulase-negative Staphylococci* and *Streptococcus dysgalactiae (Strep. dysgalactiae)*, two predominant minor and major pathogenic bacteria at three levels of SCC [~300, 800, and 1,000 (×10^3^) cells mL^−1^ for subclinical and clinical mastitis, respectively] were examined in our study. Moreover, normal milk (sample N) was set as a control expecting minute NAGase activity in which negative microbiological output was attained (no traces of any bacterial contaminants) presenting insignificant SCC value of 60,000 cells mL^−1^. All milk sampled were evaluated using optimized condition, both for TEOS modification and quantum material content. Figure 4A depicts NAGase enzymatic activity product FL values in different milk samples (normal, subclinical, and clinical mastitis) with the addition of ZnO-QDs-SiO_2_ onto the reaction assay solution. Substantial increase in FL values (*t*-test, *p* < 0.05) are shown in correlation to SCC level escalation, presenting intensities of 15.1 ± 0.1, 21.2 ± 0.5, 26.7 ± 0.6 a.u. for S1, S2, S3 (all positive with *Coagulase-negative Staphylococci* bacteria) with respect to sample N (negative control with FL value of 4.5±0.3 a.u.). Similar FL intensities tendency is shown for *Strep. dysgalactiae* pathogen (samples S4, S5, S6, *t*-test, *p* < 0.05), further strengthening the correlation between the obtained FL values (NAGase activity) within the different milk samples to the escalation of SCC levels. Milk samples S3 and S6 recognized as clinical mastitis (SCC score >1,000,000 cells mL^−1^) presented the highest emitted intensities assuming higher NAGase activity. This can be ascribed to the severity of mastitis at higher SCC scores for both analyzed pathogens resulting in higher enzymatic product concentrations within the examined milk samples (Viguier et al., 2009; Hovinen et al., 2016). These results overlap with an earlier report by Pyörälä et al. that have shown the correlation of NAGase activity to the severity of mastitis and udder health (Pyörälä et al., 2011). Figure 4B shows the conventional modified assay results which omit the use of quantum nanomaterials within the protocol assay. Non-significant differentiation is shown for mastitic milk samples at the entire SCC range for both analyzed pathogens (*t*-test, *p* > 0.05), except sample S6. The resulting limits the classification of mastitis severity or the specific clinical stage of the animal based on conventional biomarker activity assay.

**Table 1:** Infection status and SCC values of the analyzed milk samples.

**Sample**	**SCC (×10^**3**^) cells mL^**−1**^**	**Bacteria**
N	60	N/A
S1	337	*Coagulase-negative Staphylococci*
S2	821	*Coagulase-negative Staphylococci*
S3	>1,000	*Coagulase-negative Staphylococci*
S4	353	*Strep. dysgalactiae*
S5	797	*Strep. dysgalactiae*
S6	>1,000	*Strep. dysgalactiae*

*N, normal milk sample; S, mastitic milk samples*.

Next, the practical aspect of the presented assay with ZnO-QDs-SiO_2_ was applied for NAGase activity quantification, acquired under optimized conditions, in comparison to the results obtained from the conventional technique. Both assays were calibrated upon wide range of 4-MU concentrations within control milk samples, expecting increased emission values for higher target product, see Supplementary Figure S3. The fitted regression equations of the calibration curves are FL = 4.006 × (C_4−MU_) + 0.439 (*R*^2^ = 0.98) and FL = 0.629 × (C_4−MU_) + 0.007 (*R*^2^ = 0.99) for with and without the addition of ZnO-QDs-SiO_2_, respectively. The linear regression equations are used for calculating the unknown NAGase activity in milk samples within the dynamic range of the plot. Table 2 summarizes the calculated NAGase activity values for with and without ZnO-QDs-SiO_2_. Both assays support previous examinations in which the NAGase activity is increased in correlation to the severity of the occurring mastitis and SCC levels for both predominant pathogens. FL emission amplification in the presence of ZnO-QDs-SiO_2_ offers significant differentiation between healthy, subclinical and clinical mastitis (e.g., 0.13 ± 0.01, 0.89 ± 0.03, 1.24 ± 0.02, 1.69 ± 0.03 μM min^−1^ for milk samples N, S4, S5, S6, respectively) with respect to conventional methodology omitting the addition of the quantum nanomaterials (e.g., 0.26 ± 0.02, 0.74 ± 0.13, 0.87 ± 0.09, 1.30 ± 0.02 μM min^−1^ for similar milk samples, respectively). Furthermore, the causative bacteria (major or minor pathogen) has been previously reported to influence the inflammatory response and by that inducing the secretion of the target acute phase protein onto plasma or milk (Kalmus et al., 2013; Hovinen et al., 2016). It is expected that *Strep. dysgalactiae* contaminations will result in a higher response with respect to *Coagulase-negative Staphylococci* (major and minor pathogens, respectively). Indeed, increased NAGase activity are found for *Strep. dysgalactiae* than *Coagulase-negative Staphylococci* while comparing the equivalent SCC values (S4, S5, S6 in comparison to, S1, S2, S3, respectively). It should be highlighted that that both conventional and signal amplified NAGase assay cannot differentiate between the specific bacterial species (Kalmus et al., 2013; Hovinen et al., 2016). Overall, our experimental evidence for intensified fluorophore emission in the presence of optically active ZnO-QDs-SiO_2_ played a crucial role for extrapolating the conventional NAGase assay onto the subclinical state identification of the early stage inflammation onset. The early stage detection of subclinical mastitis by conventional manners is impossible for future technology adaptation for on-site or on-line sensing application without the addition of quantum nanomaterials onto the assay solution. Therefore, these results further confirm the applicability of the enhanced FL emission in the presence of optically active ZnO-QDs-SiO_2_, which offer feasible means of signal amplification and discrimination between the obtained milk qualities, and thus depicting the severity of the occurring mastitis within the herd for early diagnosis. Moreover, the addition of ZnO-QDs-SiO_2_ to the protocol assay can potentially help veterinarians assessing the mastitis recovery prognosis with higher resolution than current manners.

**Table 2:** NAGase activity in milk samples with and without the addition of ZnO-QDs-SiO_2_ onto the reaction assay.

**Sample**	**NAGase activity** **without ZnO-QDs-SiO_**2**_** **(μM min^**−1**^)**	**NAGase activity** **with ZnO-QDs-SiO_**2**_** **(μM min^**−1**^)**
N	0.26 ± 0.02	0.13 ± 0.01
S1^a^	0.60 ± 0.09	0.46 ± 0.00
S2^a^	0.66 ± 0.03	0.65 ± 0.02
S3^a^	0.83 ± 0.11	0.82 ± 0.02
S4^b^	0.74 ± 0.13	0.89 ± 0.03
S5^b^	0.87 ± 0.09	1.24 ± 0.02
S6^b^	1.30 ± 0.02	1.69 ± 0.03

*N, normal milk sample; S, mastitic milk samples*.

*Data are reported as mean ± standard deviation (n ≥ 3)*.

a *Positive with Coagulase-negative Staphylococci*.

b *Positive with Strep. dysgalactiae*.

In summary, we successfully developed a sensitive discrimination assay of subclinical and clinical mastitis based on enhanced FL emission using ZnO-QDs-SiO_2_ within the conventional NAGase activity assay, known as a golden standard methodology for biochemical mastitis evaluation. Increased NAGase activity levels indicate epithelial cell destruction within the udder generally used as a prognostic tool of mastitis. The enhanced FL emission values, over 11-folds, were obtained in the presence of optically active quantum nanomaterials, which offer milk quality differentiation in correlation to the severity of the occurring mastitis, SCC levels and the causative pathogens. The presented proof-of-concept offers an efficient, simple, cost-effective FL signal amplification for early stage mastitis onset identification and thus revealing the actual animals' clinical status. Moreover, the optoelectronic properties of the selected nanomaterial can be easily fine-tuned offering the flexibility and efficiency for enhancing the signal capabilities, dynamic range and system's resolution. Additionally, it should be highlighted that the presented assay can be personalized and applied for the study of other protein classes (i.e., predictive inflammation biomarkers) with similar optical properties upon minor assay modifications and improvements.

## Data Availability Statement

All datasets generated for this study are included in the article/Supplementary Material.

## Ethics Statement

Animal experiments were approved by the Institutional Animal Care Committee of ARO, The Volcani Center, Bet Dagan, Israel and followed its ethical guidelines (approval number 838/119IL).

## Author Contributions

GS and NN designed the study, conducted the experiments, analyzed the data, drafted, and revised the manuscript. All authors read and approved the final manuscript.

### Conflict of Interest

The authors declare that the research was conducted in the absence of any commercial or financial relationships that could be construed as a potential conflict of interest.
