# Supplementary material for: Enhanced Fluorescence of N-Acetyl-β-D-Glucosaminidase Activity by ZnO Quantum Dots for Early Stage Mastitis Evaluation
Source: Front Chem. 2019 Nov 8;7:754. doi: 10.3389/fchem.2019.00754 (PMC6856209; doi:10.3389/fchem.2019.00754)
Supplement: Supplementary file 1 [file Data_Sheet_1.docx]

Supplementary Material

Enhanced fluorescence of N-acetyl-β-D-glucosaminidase activity by ZnO quantum dots for early stage mastitis evaluation

Narsingh R. Nirala^1^, Giorgi Shtenberg^1*^

^1^ Institute of Agricultural Engineering, ARO, the Volcani Center, Bet Dagan 50250, Israel

*** Correspondence:**Dr. Giorgi Shtenberg
giorgi@agri.gov.il


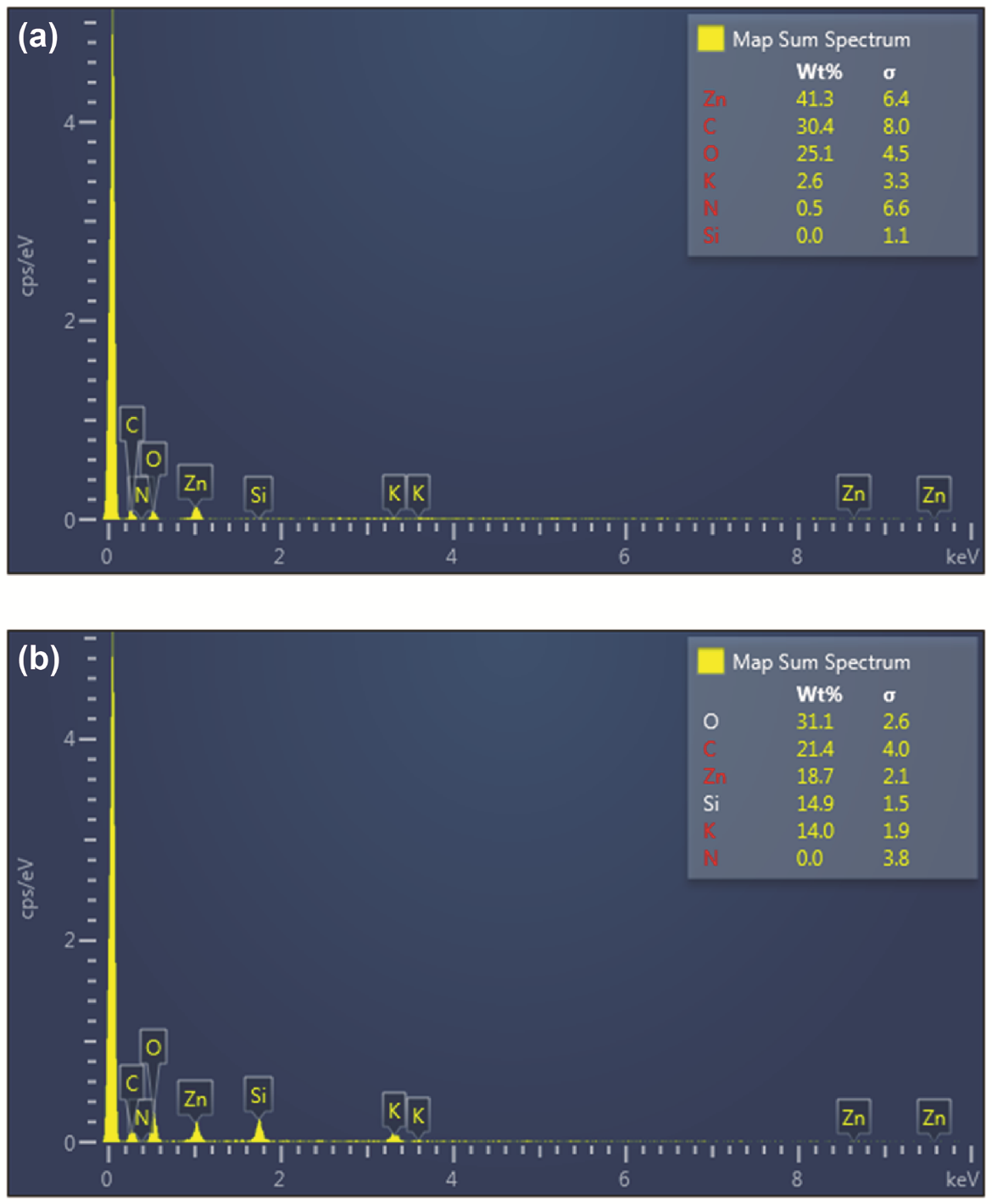


**Supplementary Figure S1.** EDX elemental analysis of (a) ZnO-QDs and (b) ZnO-QDs-SiO_2_.


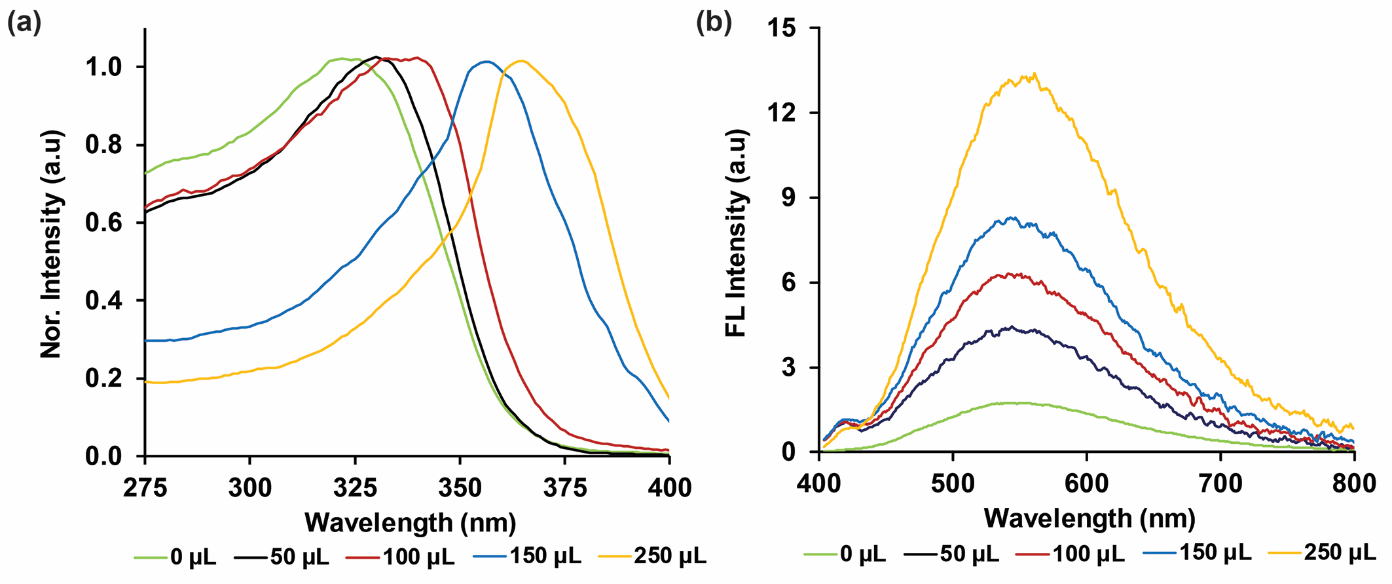


**Supplementary Figure S2.** (a) Excitation and (b) emission spectra of different TEOS amounts (0, 50, 100, 150 and 250 µL) added to coat ZnO-QDs, according to the noted synthesis protocol of ZnO-QDs-SiO_2_ under NAGase activity experimental conditions.


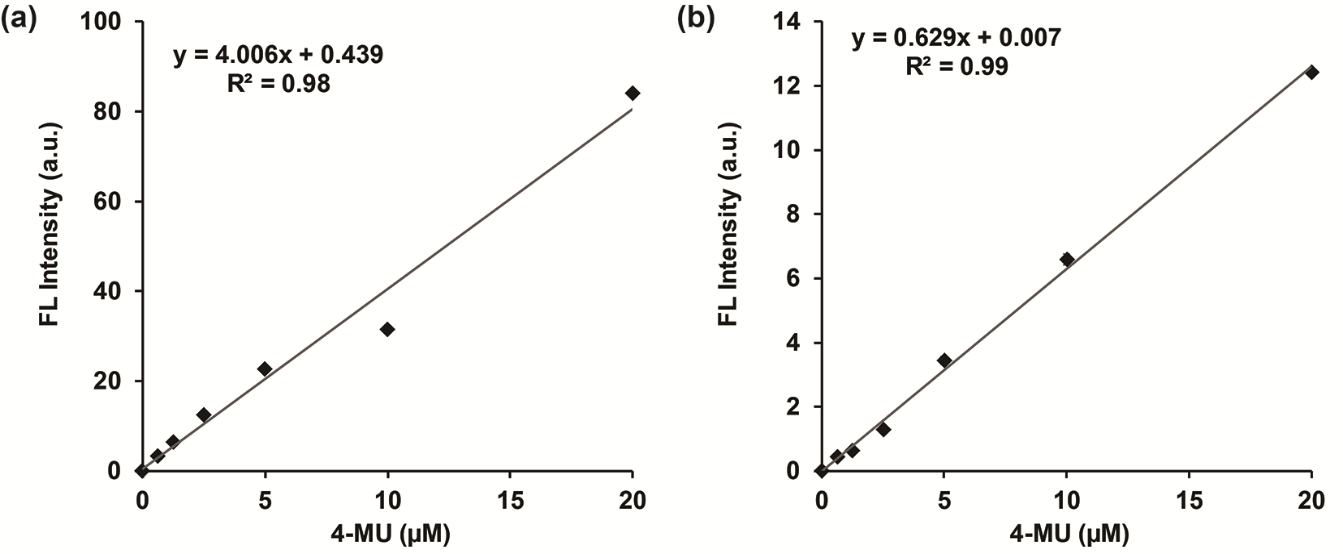


**Supplementary Figure S3.** Calibration curves of NAGase enzymatic activity product (4-MU) FL emission intensities within control milk samples (a) with and (b) without ZnO-QDs-SiO_2_ addition onto the reaction assay solution. Data are reported as mean ± standard deviation (n ≥ 3).
